# Physiotherapy in nursing homes. A qualitative study of physiotherapists’ views and experiences

**DOI:** 10.1186/s12877-021-02080-6

**Published:** 2021-03-01

**Authors:** Shanty Sterke, Ana Paula Nascimento da Cunha, Hanneke Oomen, Lennard Voogt, Marleen Goumans

**Affiliations:** 1 Research Centre Innovations in Care, Rotterdam University of Applied Sciences, Rotterdam, The Netherlands; 2 Department of Physiotherapy, Aafje Nursing Homes, Rotterdam, The Netherlands; 3 Department of Public Health, Erasmus University Medical Center, Rotterdam, The Netherlands; 4 Department of Physiotherapy, Laurens Care Center, Rotterdam, The Netherlands; 5 Department of Physiotherapy, Swinhovegroep, Zwijndrecht, The Netherlands; 6 Pain in Motion Research Group (PAIN), Department of Physiotherapy, Human Physiology and Anatomy, Faculty of Physical Education and Physiotherapy, Vrije Universiteit Brussel, Brussels, Belgium

**Keywords:** Physiotherapy, Nursing homes, Long-term care

## Abstract

**Background:**

There are distinct differences in the implementation of physiotherapeutic care in nursing homes. Both nationally and internationally staffing levels of physiotherapy differ significantly between and within nursing homes. Since legislation or guidelines that specify the parameters of physiotherapy required in nursing homes are lacking, it is unknown how physiotherapists currently estimate the usefulness and necessity of physiotherapy in individual situations in long-term care. The purpose of this study was to describe how physiotherapists actually work, and how they want to work, in daily practice in Dutch nursing homes.

**Methods:**

We performed a qualitative study with an online questionnaire. We asked 72 physiotherapists working in Dutch nursing homes to describe as accurately as possible usual care in nine different cases in long-term care. Furthermore we asked them to describe their role in the prevention and treatment of a number of indicators that measure the quality of care in nursing homes. Two reviewers thematically analysed the answers to the questionnaires.

**Results:**

Forty-six physiotherapists returned the questionnaire. Physiotherapy services include active exercise therapy aimed to improve mobility and movement dysfunctions, advising on prevention and management of falls, pressure ulcers, incontinence, malnutrition and sarcopenia, overweight, physical restraints, intertrigo, chronic wounds, behavioural and psychological symptoms in dementia, and physical inactivity, and ergonomic and behavioural training. The way and extent in which physiotherapists are involved in the various care- and functional problems differs and depends on organisational and personal factors such as, organisation’s policy, type of ward, time pressure, staffing level, collaboration with other members of the multidisciplinary team, or lack of knowledge.

**Conclusion:**

Physiotherapists in nursing homes are involved in the prevention and management of different care situations and functional problems. The way in which they are involved differs between physiotherapist. Aiming for more uniformity seems necessary. A shared vision can help physiotherapists to work more consistently and will strengthen their position in nursing homes.

## Introduction

The number of old and very old people in the world increases and challenges health care systems to provide effective and sustainable care [1]. In The Netherlands, approximately 8 % of the people aged 65 years and older live in a nursing home. It is estimated that the number of people living in institutional care facilities will increase in the years to come [2].

Residents in nursing homes are often frail and usually depend on integrated care in which the nursing staff, physicians, physiotherapists, occupational therapists, and other care providers work together in order to provide optimal support [3].

Physiotherapists are involved in the multidisciplinary care for residents with pain [4], pressure ulcers [5, 6], wound care [7], urinary incontinence [8], and/or falls [9]. Outcomes of physiotherapy interventions can be on a resident level (e.g., ADL functioning), or on a facility level (e.g., monthly falls rate, education to staff, environmental changes, facility policies) [10, 11]. As a consequence, physiotherapists in nursing homes provide care for different sorts of specific and complex health problems under local circumstances and financial, supportive and institutional constraints. This may lead to distinct differences in the implementation of physiotherapeutic care in nursing homes, a situation that is currently reported by several research groups. In addition, recent studies described that both nationally and internationally staffing levels of physiotherapy significantly differ between and within nursing homes [12–15].

A previous study showed a significant, positive relationship between physiotherapy staffing levels and quality of care. Evidenced by quality measures as the number of fall injuries, the percentage of residents with moderate to severe pain, pressure ulcers, functional decline [14].

We assume that staffing level will influence the choices physiotherapists make in daily practice. Moreover, as far as we are aware, clear guidelines for physiotherapy in nursing homes are lacking. Therefore, legitimizing evidence based interventions, demarcating professional domains and roles within specific local (institutional) circumstances and within interprofessional teams are inherent and complex aspects of physiotherapy in this health care context.

Since there are no guidelines or legislation that specify the parameters of physiotherapy required in nursing homes, we think it is important to understand how physiotherapists currently estimate the usefulness and necessity of physiotherapy in individual situations in long-term care. Reviewing the current situation is the first step towards the development of a quality standard, in which a shared vision of how physiotherapists in nursing homes are both currently working, and want to work is documented.

The aim of this study is to describe the methods of working of physiotherapists in daily practice in Dutch nursing homes, and how physiotherapists want to position themselves within this context.

## Methods

### Study design

We performed a qualitative study with an online questionnaire. We invited all physiotherapists working in nursing homes in an urban area (Rotterdam-Rijnmond) in The Netherlands. We also approached physiotherapists from the healthcare organisations that collaborate in the University Network for the Care sector Zuid-Holland (UNCZH) and colleagues from the researchers’ personal networks, to participate. In addition, we made an appeal for participants in the newsletter of the Dutch association for geriatric physiotherapy.

Most nursing homes in the Netherlands have separate wards for long-term physical care, and for residents with psychogeriatric problems. Physiotherapists who provide specific paramedical care for older people permanently living on these long-term care wards were included. Exclusion criteria were physiotherapists who work exclusively on short-term care wards and/or on geriatric rehabilitation wards, where residents stay temporarily and will be expected to be discharged.

The participants received an online questionnaire in which we asked them to describe their role in the prevention and treatment of common care and functional problems in daily practice in the nursing home.

### Questionnaire

#### Design

In order to develop the questionnaire, we asked an expert panel of six physiotherapists to discuss with us which topics should be covered in the questionnaire, and which cases and scenarios are a good reflection of daily practice. The experts were geriatric physiotherapists specialised in the care for older patients, or had an extensive practical experience in nursing home care. The expert panel had on average 22,5 (± 8,4) years of experience in nursing home care.

We tested the pilot questionnaire in this expert panel. The questionnaire was repeatedly modified in an iterative process with the expert panel, until consensus was reached.

#### Content

The questionnaire consisted of two parts, both with open questions. In the first part we asked the physiotherapists to describe as accurately as possible the care as usual in nine different cases in long-term care. In describing the cases, we made a distinction between psychogeriatric and somatic problems, and we took into account that cognitive and physical problems occur in different degrees (Table 1).

**Table 1:** Cases in the first part of the questionnaire

Case	Diagnoses	Physical and cognitive functioning
1	Chronic Obstructive Pulmonary Disease (COPD)/ heart failure/cox- and gonarthrosis.	- increased need for care - walks independently with a walker - balance problems and an increased risk of falling
2	Dementia	- moderate to severe cognitive decline - needs stimulation, instructions and help with the activities of daily living (ADL) - goes to the toilet independently - walks independently with a walker, occasionally forgets it or uses it incorrectly
3	Dementia	- very serious cognitive decline - restlessness and wandering behaviour - frequent faller
4	Cerebrovascular accident	- can walk with support of the nursing staff - regularly makes a wrong assessment when moving, for example, threatens to sit down next to the chair
5	Dementia/ readmission from the hospital after fall resulting in hip fracture	- moderate to severe cognitive decline - Before the fall needed stimulation, instructions and help during the ADL, walked independently with a walker, occasionally forgot it or used it incorrectly. Went to the toilet without any assistance - Since readmission sitting in a wheelchair and can start rehabilitation.
6	Lower leg amputation/ diabetes/ vascular disease	- continent - needs help with an active transfer aid to go to the toilet - the transfer with active transfer aid is becoming more and more difficult
7	Dementia	- severe cognitive decline - agitation, i.e., inner restlessness leading to purposeless and highly repetitive motor activity - needs help to go to the toilet with an active transfer aid - moves his feet during the transfer, which is dangerous, he could fall
8	Cerebrovascular accident	- permanently sitting in a wheelchair.
9	Parkinson’s disease/ dementia	- very severe cognitive decline - permanently sitting in a wheelchair - high tonus.

For each case, we asked the physiotherapists to describe what they actually do in their current work by the following questions:

1. Describe your involvement shortly after the admission into the nursing home, in the first few weeks.
2. Describe your involvement after the first weeks, during the following months and years.
3. Describe your involvement in the palliative phase.
4. Can you do your job the way you want? If not, which barriers do you face?

We asked them to consider both directly client-related and indirectly client-related activities, such as the purchase of aids; contact with other people and/or organisations; consulting or advising other disciplines or family members.

The second part of the questionnaire consisted of general questions about the prevention and treatment of a number of indicators that measure the quality of care in nursing homes [16]. Measuring the prevalence, prevention, and treatment of quality indicators offers healthcare organizations the opportunity to improve their quality of care [17]. Furthermore, quality measures are topics of interest for external supervision by the health care inspectorate [18]. These quality measures were: pressure ulcers, incontinence, malnutrition, overweight, falls, physical restraints, intertrigo, chronic wounds, transfers, behavioural and psychological symptoms in dementia (BPSD) and physical inactivity. For each quality measure, we asked the following two questions:

1. What is the policy regarding prevention and treatment of this quality measure in your institution?
2. What role do you have, or would you like to have, in the prevention or treatment of this quality measure?

### Ethical approval

Research with online questionnaires filled out by healthcare providers does not require approval of an ethics review board in The Netherlands. Therefore, the study protocol was not examined in an ethics committee. All participants gave their informed consent. All study data were handled confidentially and in accordance with the Personal Data Protection Act. Only the first author (CS) had access to the questionnaires. To ensure the security of the data and the protection of the participants’ privacy, all participants were coded by a random two-figured number. Besides CS, nobody had access to the code.

#### Analysis

Two researchers (CS and HO) read the answers of the physiotherapists to the questionnaire separately, segmented the text into meaningful expressions, and described the fragments in a single word or short sequence (open coding). In a meeting, the two researchers discussed their individual analysis to reach consensus about the codes. Next, the coded text fragments were related to each other by first combining the fragments with similar codes and assessing which meanings could be summarised under a new code (axial coding). Finally, the two researchers discussed how these axial codes were related to each other. Based on the content of identified themes and the interrelationships between them, we formulated an answer to the research question of this study.

## Results

Seventy-two physiotherapists had signed up to participate in the study. Forty-six of them returned the questionnaire. The 26 physiotherapists that did not return the questionnaire worked in twenty different nursing homes. We have no further data from the non-respondents. The respondents worked on average 20.3 (± 9.5) hours a week on a long-term care ward in a nursing home, delivering care to 55 (± 29.3) residents. The physiotherapists had on average 13.8 (± 10.9) years of experience in long-term care.

The answers of the respondents can be summarised in four themes, namely: care and functional problems; the way the physiotherapist gets a referral; the observations, assessments, physical examinations and analyses; physiotherapy interventions and actions.

### Care and functioning problems

The way and extent in which physiotherapists are involved in the various care and functional problems differs. Whether a therapist is involved or not, depends on organisational and personal factors. For example, organisation’s policy, type of ward, time pressure, staffing level, collaboration with other members of the multidisciplinary team, or lack of knowledge.

With regard to the prevention and management of pressure ulcers, 37 respondents are involved. They evaluate and advise on lying and sitting posture, footwear, and anti-decubitus materials. Twenty-nine of them work in close contact with the occupational therapist on this problem, and with a large overlap of tasks. Those who are not involved, think that pressure ulcers are the occupational therapist’s field of expertise.

Nine physiotherapists are involved to manage or prevent incontinence. They give exercise therapy in order to keep the transfer to the toilet active as long as possible, and exercises to strengthen the pelvic floor muscles. Furthermore, they advise on sitting posture on the toilet and good use of toilet enhancers. Five respondents want to be involved. One of them thinks that the specialist knowledge to deal with this care problem is lacking, and that physiotherapists need further education on the pelvic floor muscles.

Four physiotherapists are involved in the management or prevention of malnutrition, and nine want to be more involved. They (want to) work closely together with dietitians, to match nutrition and physical training, especially in cases of severe sarcopenia or in educating the nursing staff on sarcopenia.

In case of overweight, nineteen physiotherapists have a role in advising, stimulating and guiding residents to physical activity, in close contact with the dietician. Furthermore, ergonomic training of the nursing staff, and purchase of aids are also tasks carried out by physiotherapists. Eight respondents want to be involved.

Most (*n* = 33) physiotherapists are involved in falls prevention. They mention directly client-related activities, such as fall risk assessments; balance and strength training; providing walking aids. Furthermore, they are involved in activities on an organizational level, such as participating in a falls prevention team, and educating staff.

Twenty-eight respondents are involved in the management of physical restraints. Thinking along in solutions with the other members of the multidisciplinary team to limit the use of physical restraints as much as possible, advising on alternatives. One respondent would like to give more education about preventing physical restraints.

With regard to the management of intertrigo, only six respondents are involved. They advise on applying the Passivity of Daily Living (PDL) method. This is an interdisciplinary approach comprising a complex of actions, facilities and measures to optimally guide, care for and nurse ‘passive’ patients. E.g., advise on lying or sitting posture, or teaching haptonomic techniques during daily care in order to prevent pain and restlessness, defensive tension and contractures [19].

Eighteen respondents are involved in the management of chronic wounds. They mention advise on lying and sitting posture, and physical activity, contact with the orthopaedic technician, and management of oedema.

Almost all (*n* = 42) physiotherapists give transfer advices for individual residents and/or general ergonomic training in transfer techniques.

Sixteen respondents are involved in the management of BPSD. These are mainly physiotherapists who had followed a course in Sensory Integration (SI). The therapist observes the interaction between senses and purposive movement, and analyses how a resident with BPSD behaves and process sensory stimuli. Responsive behaviours can result from over-stimulation or under-stimulation. A respondent expressed it as follows: ‘I think that a lot of responsive behaviours come from the body. Too much or too little stimulation, uncomfortable lying or sitting, pain, sleep disturbances. The physiotherapist can influence all these factors’.

Eighteen respondent are involved in promoting physical activity. They have an active role in advising, and educating family members, volunteers, and other staff. Furthermore, by participating in committees, they also have an active role in the promotion of physical activity at the facility level.

### Referral

Depending on the case, one (cases 5, 7 and 9) to nine (case 1) respondents mention that they see a newly admitted resident only on referral of a physician. Others see newly admitted residents without a specific referral. They introduce themselves to the resident and his or her family members, examine whether there is a request for help in the domain of physiotherapy. Furthermore, they assess the functional mobility and fall risk, inspect the footwear, arrange new footwear and walking aids if necessary, for each new resident. One respondent thinks it’s important to assess the mobility of every newly admitted resident, however it depends on how busy it is. ‘Normally, I will introduce myself and get acquainted as soon as possible. In busy periods, I am waiting for a request for advice from the doctor or from the nursing staff’.

After the first few weeks of the admission to the nursing home, physiotherapists become involved either on referral or unsolicited. In case 3, twenty-nine respondents mention that they come into action only on doctor’s referral or when they receive a specific question from the resident, a relative or caregiver, or from the nursing staff or a colleague from another discipline in the multidisciplinary team. In the other cases this varies between zero tot six respondents.

When there is no specific defined treatment goal, or no specific request or referral, after the first few weeks, some physiotherapists walk around the ward in order to continue monitoring possible care problems. Eighteen respondents in case 2, thirteen respondents in case 3, and ten in case 1. In the other cases this varies between one and nine respondents. In their opinion, care and functioning problems can be prevented by acting pro-active. One respondent said: ‘I would like to monitor on a regular basis how people are functioning. Now I’m dependent on the nursing staff, who first make their own decisions about whether or not my involvement is necessary. Often you catch sight of a resident pretty late. It then takes a lot of energy to get back to a better level. For instance, when people develop wounds, I would like to be involved timely so that we can improve blood circulation and possibly reduce edema so that wound healing improves’.

### Examinations and assessments

There are several ways in which physiotherapists make the assessments of the care and functional problems. At first, they use standardized clinical measurements to assess physical functioning. They mentioned specific measurements such as the Berg Balance Scale, Timed-Up-And-Go Test, Visual Analogue Scale, Functional Ambulatory Categories, Elderly Mobility Scale, Performance Oriented Mobility Assessment, Medical Research Council dyspnoea scale, 10 Meter Walk Test, 2-Minute Walk Test, 6-Minute Walk Test, Medical Research Council Scale, Hand Held Dynamometer, Timed Chair-Stand-Test, Romberg test, MINI-Best test, motricity Index, Frenchay Arm Test, Patient-Specific Complaints, Passivity’s of Daily Living list, Paratonia Assessment Instrument, gait analysis, and oxygen saturation.

In addition a large part of the work consists of observing both residents and nurses without a specific instrument during activities of daily living. For instance, in case 3 (*n* = 18) and case 7 (*n* = 28) physiotherapists observe the resident to find out which stimulus triggers the restless behaviour, and if patterns can be discovered. They try out different strategies to reduce the restlessness. They observe the physical capability of the resident, and the way other people interact with the resident. One respondent answered to case 7: ‘As a physiotherapist, I would focus on analysing the transfers. Why does he move his feet during the transfer? Is it possible that he can do more than the nurses think? Is he able to make the transfer actively himself under supervision with or without a walking aid? Does the problem with the transfer depend on how the nurses relate to the resident? Does it depend on the instruction he receives? Or is it the agitation, restlessness and purposeless behaviour? Is there a need for exercise that, if fulfilled, makes the resident calmer’.

### Interventions

Whether a physiotherapy intervention consists of exercise therapy depends on the case. In case 1, 24 respondents mention to start therapy to improve balance, endurance, and strength. Is case 2, twelve physiotherapists start therapy to improve walking function or quadriceps strength. Three respondents practice correct use of the walker. All other respondents in this case mention to give a tailor-made advise to the nursing staff and the family members to reduce fall risk, such as optimizing footwear, and how to use the walker correctly. In case 3, only one respondent mentioned to start therapy aimed reduce falls by improving dynamic balance and gait function. All others advise on fall risk reduction, footwear, walking aids. In case 4, twenty-four physiotherapists start therapy after they judged that the resident can improve. They use motor learning techniques to improve a safe transfer. One respondent answered to case 4: ‘physiotherapy trajectories always have a beginning and an end. Physical activity to maintain the level of functioning is not my department. That the job of the activity coach, whom I advise and guide where necessary’. There are also therapists who feel that exercise therapy to maintain the level of physical functioning is a part of their job. As one therapists said: ‘I’d like to offer residents more exercises. Many residents in long-term care are inactive, which has negative consequences. Because of this, I often feel that I am more busy putting out fires than actually preventing physical complaints or maintain the level of physical functioning’. In case 5, 32 respondents mention that therapy is aimed to restore physical functioning to the level before the hip fracture by improving walking distance, endurance, strength, and transfers. In case 6, 31 respondents start therapy to maintain standing function, strength, quadriceps strength, endurance, balance, and coordination. In case 7, four respondents, start active therapy aimed to improve the transfers, or offer a physical exercise plan to maintain physical functioning. In case 8, three respondents assess if the resident’s physical function can improve. Seventeen respondents start therapy to maintain the level of physical functioning by offering muscle strength and balance exercises. In case 9, four therapist start controlled active motion therapy, or MOTOmed movement therapy to prevent joint contractures, and decrease muscle tone. All other therapists evaluate lying and sitting posture in case 8 and case 9, in order to provide adjustments to increase sitting and lying comfort.

The physiotherapist’s involvement in the palliative phase is the same in all cases. Namely, interventions aimed at increasing comfort, such as advices on lying and sitting posture, transfer techniques and prevention of pressure ulcer. Furthermore, relaxation exercises are mentioned.

All therapists that finish the exercise therapy encourage residents to physical activity self-management, or delegate the activities to an exercise coach, an activity coach, the nursing staff, a volunteer or carer. However, some physiotherapists indicate that residents, or their carers have different expectations from the physiotherapy. For example, there are residents who would like to use the mechanized movement trainer in the physiotherapy gym in the presence of the physiotherapist. There is an area of tension between the physiotherapists’ opinion and the residents’ wishes. Because exercise is not synonymous with physiotherapy. Therefore, in the physiotherapists’ opinion, any other person can support or supervise the resident in this case. One physiotherapist said: ‘some people keep on asking for this. But because I am a physiotherapist, and no movement therapist, I am not involved in just physical activity to maintain physical functioning. I haven’t been doing this for years and I don’t see any complications in leaving out this way of performing physiotherapy. Selling no to purposeless cycling on a movement trainer or walking between bars, can be a challenge’.

Besides all the direct activities on a resident level, all respondents reported that physiotherapists play a role on the organisation level or the facility level. They are members of one or more committees. Such as a falls prevention team, pressure ulcer committee, or physical work load committee. Their role is to participate in policy making or decision making on the purchase of materials. For instance anti-decubitus materials and transfer aids. Also on educating nursing staff, family members, or volunteers on falls prevention, physical activity, transfer techniques. On a ward level physiotherapists play an important role in falls prevention activities.

## Discussion

Physiotherapists are involved in the prevention and management of care and functional problems. They are helping to slow down functional decline, and improve functional ability and quality of life. Physiotherapy services include active exercise therapy aimed to improve mobility and movement dysfunctions, advising on prevention and management of care problems, falls prevention, and ergonomic and behavioural training. The way and extent to which physiotherapists are involved differs. This is due to local policy or lack of time. There are also different points of view about the interpretation of the profession.

An earlier study showed that there is no ‘one-size fits all’ approach to enhancing evidence based practice implementation in physiotherapy. The organisational culture seems to play a crucial role in implementing evidence based interventions [20]. Differences in culture between organisations may be one of the reasons why we found different working methods between the therapists in our study. For instance, in some organisations, a physicians’ referral is necessary for the physiotherapist to go into action. This can help to sort out the requests for help and unburden the physiotherapist. On the other hand, involvement without a specific referral can be interpreted as a proxy of the usual involvement of the physiotherapists within the multidisciplinary team. Moreover, evidence for the effect of physiotherapy in long-term care is moderate. Effects of physical rehabilitation for long-term care residents appear to be quite small and may not be applicable to all residents. There is also insufficient evidence that improvements sustain or which interventions are most appropriate [21]. However, conclusive scientific evidence does not do justice to the personal character of good care in nursing homes.

Traditionally, physiotherapy is examined from a theoretical framework, in which improving physical outcome measures (e.g. strength, endurance, balance, walking speed) or pain reduction serve as a starting point. The assessed outcome measures are often irrelevant to physiotherapy in long-term care. In addition, the interventions studied mostly concern standard programmes in heterogeneous research populations [21]. This is not in accordance with daily practice in a nursing home where individual situations strongly differ, and where physiotherapists are involved in unique and complex care problems [22].

Physiotherapists are mainly focused on optimizing daily life by considering the residents’ possibilities to maintain (or improve) daily functioning. Based on the expected possibilities of the resident, the physiotherapists can give exercise therapy in order to reach an achievable goal. However, in their daily work they do much more than just give exercise therapy. On the basis of extensive observations of residents in daily life, they formulate advices on a variety of issues relating to mobility, posture, and movement dysfunctions. Physiotherapists largely integrate the therapy into everyday life. There they are in close contact with the family and the nursing staff. They constantly work in close contact with the other members of the multidisciplinary team. The multidisciplinary approach, in which the physiotherapist plays a unique role, has been found important in an international Delphi study on the assessments and management of geriatric syndromes [23].

Nursing home residents often suffer from multi-morbidities and geriatric syndromes [24]. Geriatric syndromes involve multiple organ systems and represent clinical conditions that share underlying causative factors [25, 26]. Examples of geriatric syndromes include incontinence, cognitive impairment, falls, impaired mobility, malnutrition, weight loss and functional decline [27–29]. In a recent study in over 12,000 nursing homes in the United States researchers analysed data from various databases, such as annual state inspection surveys and certification processes. They studied the relationship between physiotherapy/occupational therapy staffing levels and a number of quality indicators. Such as: the percentage of residents requiring more help in their activities of daily living since the last quality measurement; the percentage of residents with severe fall injuries; the percentage of residents with moderate to severe pain; pressure spots; a physical restraint; anxiety or depression; incontinence; a catheter; urinary tract infection; weight loss. This study found that nursing homes that had the highest physiotherapy/occupational therapy staffing level, were on average, independently of the nursing staff level, associated with more favourable performance scores on the quality measures, compared to facilities with lower staffing levels [14].

This is in line with the physiotherapists’ opinions in our study. Physiotherapists provide benefits to residents in certain quality domains that nursing staff have less of an influence on. Livingstone et al. (2019) do not distinguish physiotherapy from occupational therapy [14]. Also in our study physiotherapists and occupational therapists work closely together, and there is a large degree of overlap between physiotherapy and occupational therapy. Therefore, it’s difficult to demarcate the professional domain and role.

The fact that there are differences in the way of working between physiotherapists, and in staffing levels between organisations may be due to the fact that there seems to be a lack of efficient documentation processes (e.g. information technology programs) and use of evidence-based benchmarks to monitor physiotherapy services. A recent systematic review aimed to consider how physiotherapy services were documented and monitored in nursing homes found that none of the included studies provided data on this topic [12].

A strength of this study is that in formulating the cases, we took into account the heterogeneity of the nursing home population. As a result, we think we have covered the care and health problems in long-term care quite well.

A potential limitation of this study is that the context is specific to Dutch nursing homes. However, we think that our results are also relevant for other countries. Because physiotherapy in nursing homes is not a typical Dutch service. Worldwide there is attention for the benefits of physiotherapy in falls prevention, pain management, improvement in physical performance, well-being, and functional ability, on pressure ulcer management in nursing homes [6, 30–33].

Another potential limitation of this study is that there may be some selection bias. From the 72 physiotherapists that had signed up to participate, 46 returned a completed questionnaire. However, since the answers were very diverse and the therapists worked in different organisations and differed in years of experience and age, we think that we surveyed a reasonably diverse group of therapists.

Furthermore, we only asked the physiotherapists’ opinions. We have no information about the staffing levels and opinions of the other members of the multidisciplinary team. Since there is some overlap between the different disciplines, especially with occupational therapy, there is no clear difference between what does and doesn’t belong to the domain of physiotherapy. Besides, physiotherapists’ involvement could be influenced by the availability of other health professionals involved in each nursing home. For example, when movement therapists or occupational therapists are not available, physiotherapists are more likely to be involved in specific or unusual activities. We don’t have data about the number of other health professionals nor about the ratio of physiotherapy staffing levels and other professionals’ staffing levels.

Insufficient evidence that training effects sustain for nursing home residents [21] can be used by managers and policy makers as an argument that physiotherapy has no value in long-term care. However, our study shows that active exercise therapy is only a part of physiotherapy in nursing homes. Although, hardly any research has been done into all the other tasks and activities that physiotherapists perform in long-term care [12], the benefits seem quite clear. Managers and policy makers should take this into consideration when they have to weigh staffing levels and reimbursements of physiotherapy in nursing homes against quality of care.

Differences in insight can be addressed if the professional group records a shared vision in a quality standard. This should describe what they do and how they want to work, together with evidence-based support. This can help physiotherapists to work more consistently. It can also serve as a guide to explain to nursing home residents and their family members what they can expect from a physiotherapist. Furthermore, a standard can also help in negotiating staffing levels and reducing large differences between institutions.

In conclusion, physiotherapists in nursing homes are involved in the prevention and management of different care and functional problems. The way in which they are involved differs between physiotherapist. Aiming for more uniformity seems necessary in order to strengthen the position of physiotherapists in nursing homes.

## Data Availability

The dataset used and analysed in this study is available from the corresponding author on reasonable request.

## Abbreviations

ADL: Activities of daily living
UNCZH: University Network for the Care sector Zuid-Holland
PDL: Passivity of Daily Living
BPSD: behavioural and psychological symptoms in dementia
SI: Sensory Integration
